# Clinical, Serological, Whole Genome Sequence Analyses to Confirm SARS-CoV-2 Reinfection in Patients From Mumbai, India

**DOI:** 10.3389/fmed.2021.631769

**Published:** 2021-03-09

**Authors:** Jayanthi Shastri, Swapneil Parikh, Sachee Agrawal, Nirjhar Chatterjee, Manish Pathak, Sakshi Chaudhary, Chetan Sharma, Akshay Kanakan, Vivekanand A, Janani Srinivasa Vasudevan, Ranjeet Maurya, Saman Fatihi, Lipi Thukral, Anurag Agrawal, Lancelot Pinto, Rajesh Pandey, Sujatha Sunil

**Affiliations:** ^1^Kasturba Hospital for Infectious Disease, Mumbai, India; ^2^International Centre for Genetic Engineering and Biotechnology, New Delhi, India; ^3^Council of Scientific and Industrial Research-Institute of Genomics and Integrative Biology (CSIR-IGIB), New Delhi, India; ^4^P. D. Hinduja Hospital, Mumbai, India

**Keywords:** SARS-CoV-2, COVID-19, reinfection, whole genome sequencing, seroconversion

## Abstract

**Background:** SARS-CoV-2 infection may not provide long lasting post-infection immunity. While hundreds of reinfections have reported only a few have been confirmed. Whole genome sequencing (WGS) of the viral isolates from the different episodes is mandatory to establish reinfection.

**Methods:** Nasopharyngeal (NP), oropharyngeal (OP) and whole blood (WB) samples were collected from paired samples of four individuals who were suspected of SARS-CoV-2 reinfection based on distinct clinical episodes and RT-PCR tests. Details from their case record files and investigations were documented. RNA was extracted from the NP and OP samples and subjected to WGS, and the nucleotide and amino acid sequences were subjected to genome and protein-based functional annotation analyses. Serial serology was performed for Anti-N IgG, Anti- S1 RBD IgG, and sVNT (surrogate virus neutralizing test).

**Findings:** Three patients were more symptomatic with lower Ct values and longer duration of illness. Seroconversion was detected soon after the second episode in three patients. WGS generated a genome coverage ranging from 80.07 to 99.7%. Phylogenetic analysis revealed sequences belonged to G, GR and “Other” clades. A total of 42mutations were identified in all the samples, consisting of 22 non-synonymous, 17 synonymous, two in upstream, and one in downstream regions of the SARS-CoV-2 genome. Comparative genomic and protein-based annotation analyses revealed differences in the presence and absence of specific mutations in the virus sequences from the two episodes in all four paired samples.

**Interpretation:** Based on the criteria of genome variations identified by whole genome sequencing and supported by clinical presentation, molecular and serological tests, we were able to confirm reinfections in two patients, provide weak evidence of reinfection in the third patient and unable to rule out a prolonged infection in the fourth. This study emphasizes the importance of detailed analyses of clinical and serological information as well as the virus's genomic variations while assessing cases of SARS-CoV-2 reinfection.

## Introduction

In December 2019, a novel coronavirus (n-CoV-19) sparked an outbreak in Wuhan, China. This virus was subsequently named SARS-CoV-2 and the disease COVID-19. On 11th March 2020, there were 1,18,000 cases in 114 countries with 4,291 deaths and the World Health Organization (WHO) declared that COVID-19 was a pandemic (1).

In August, the first report of reinfection by a phylogenetically distinct strain of SARS-CoV-2 was confirmed in Hong Kong (2) and subsequently Nevada reported a confirmed reinfection in USA (3). While there have been many reports of putative reinfections based on RT-PCR positivity, this has been confounded by prolonged shedding of viral RNA in the absence of replication competent virus (4) which can continue to cause RT-PCR positivity for up to at least 83 days (5). Nevertheless, the samples from the two episodes can be sequenced and genomic analysis may demonstrate genetic variation that can't be explained by short term *in vivo* evolution, which when combined with epidemiological and clinical evidence, may confirm reinfection (2, 3).

The present study was undertaken using samples collected from individuals tested for SARS-COV-2 as standard of care either for contact tracing or diagnostic purposes in symptomatic individuals. We report a case series of four individuals who had asymptomatic or mild RT-PCR proven COVID-19 followed by a second symptomatic RT-PCR positive episode with lower Ct values and varying degrees of increased clinical severity in the second episode.

## Materials and Methods

### Study Design and Participants

We identified four individuals who had tested RT-PCR positive for SARS-CoV-2 between April to June 2020 and who tested RT-PCR positive for SARS-CoV-2 once again between July to September after presenting with symptoms suggestive of COVID-19. Based on the RT-PCR results and clinical presentation of the patients, we suspected reinfection with SARS-CoV-2. Upon confirmation of the RT-PCR findings, whole genome sequencing was performed on the stored paired samples. Clinical findings and investigations were retrieved from their case records. Blood samples were collected prior to and after the second episode for anti-SARS-CoV-2 serology including anti-N, anti-S1 RBD, sVNT (surrogate virus neutralization test). The study was approved by the Institutional Review Board of Kasturba Hospital of Infectious Diseases; IRB number 015/2020. The patients provided written informed consent.

### Procedures

#### Sample Collection

Nasopharyngeal (NP) and oropharyngeal (OP) samples for SARS-CoV-2 RT-PCR were collected, aliquoted and stored for future use as detailed in the Supplementary Table 1. Phlebotomy was performed and blood was collected in dipotassium EDTA tubes for anti-SARS-CoV-2 serology at time points between the first and second episode, early in the second episode and a longitudinal sample as described in Table 1.

**Table 1:** Clinical course, RT-PCR, and serology.

**Patient nomenclature**	**Date of RT-PCR**	**Clinical features and duration of illness**	**Ct values**			**NC CLIA IgG**	**S1 RBD CLIA IgG**	**sVNT >20% positive <20% negative**
			**N gene**	**ORF1ab**	**S gene**			
Patient A	+ve 15/5/20 –ve 19/05/20	Sore throat, nasal congestion and rhinitis. Symptoms resolved in 2 days	32	32	Nil	01/07/2020 negative 0.02	–	–
Patient A f/u	+ve 19/7/20 –ve 29/7/20	Myalgia, fever, non-productive cough, fatigue. Symptoms resolved in 1 week	25	23	23	23/7/2020 negative 0.05 16/9/2020 positive 3.32	23/07/2020 non-reactive 0.02 16/9/2020 reactive >20.00	23/07/2020 positive 25% 16/9/2020 positive 93%
Patient B	+ve 15/5/20 –ve 18/5/20	None	33	Nil	32	01/07/2020 negative 0.05	–	–
Patient B f/u	+ve 18/7/20 –ve 25/7/20	Myalgia, malaise. Symptoms resolved in 2 days	36	38	Nil	23/7/2020 negative 0.02 16/9/2020 negative 0.02	23/07/2020 non-reactive 0.00 16/9/2020 non-reactive 0.01	23/07/2020 negative 12% 16/9/2020 positive 22%
Patient D	+ve 14/5/20 –ve N/A	Sore throat, rhinitis and myalgia. Symptoms resolved in 5 days	32	34	35	4/6/2020 negative 0.04	4/6/2020 non-reactive 0.03	4/6/2020 negative 11%
Patient D f/u	+ve 7/7/20 –ve N/A	Fever, myalgia, rhinitis, sore throat, non-productive cough and fatigue. Prolonged course, unable to return to work for 3 weeks	17	18	21	8/7/2020 positive 1.4 17/9/2020 positive 2.44	8/7/2020 reactive 2.37 17/9/2020 reactive 6.39	8/7/2020 positive 60% 17/9/2020 positive 91%
Patient E	+ve 20/04/20 –ve 23/04/20	Fever, myalgia, dry cough. Symptoms resolved in 1 week	31	31	−31	02/09/2020 negative 0.03	02/09/2020 non-reactive 0.01	02/09/2020 negative 6%
Patient E f/u	+ve 04/09/20 –ve 18/09/20	Fever, myalgia, dry cough, nausea, abdominal pain, breathlessness on exertion. Prolonged source, unable to return to work for 6 weeks. Breathlessness on exertion and fatigue persisted	22	22	22	18/09/2020 positive 3.62	18/09/2020 reactive 1.91	18/09/2020 positive 74%

*Nil = not detected.

#### RT-PCR

One of the aliquots was used for RNA extraction and tested by multiplex real time RT-PCR TaqPath™ COVID19 RTPCR kit for the qualitative detection of nucleic acid of SARS-CoV-2 from Applied Biosystems. Additional details of RT-PCR testing are described in Supplementary Table 1.

#### Serology

Anti-N protein IgG antibodies were tested by qualitative ARCHITECT chemiluminescence microparticle immunoassay (Abbott Laboratories, USA). Anti-S1 RBD antibodies were tested using SARS-CoV-2 Total antibody test on Atellica IM analyzer (Siemens, Germany). Neutralizing antibodies were tested by SARS-CoV-2 Surrogate Virus Neutralization test (GenScript USA, Inc).

#### Whole Genome Sequencing

Extracted RNA from all four paired stored samples was transported at −80°C for whole genome sequencing. Sample preparation, sequencing, and data analysis was performed by previously published protocols (6). Briefly, double-stranded cDNA was synthesized from 50 ng of total RNA for all the SARS-CoV-2 positive samples. The first strand of cDNA was synthesized using Superscript IV followed by RNA digestion with RNase H for second strand synthesis using DNA Polymerase I Large fragment (Klenow fragment). One hundred nanograms of purified double-stranded cDNA for both pools of ARTIC tiling PCR primers (V3 Primer pools) were taken forward. Post-amplification, pool 1 and 2 amplicons were pooled and purified using 1x AMpure beads (AMPure XP, Beckman Coulter, Cat. No. A63881). Further, 200 ng of each purified sample of multiplexed PCR amplicons obtained was taken for library preparation using Oxford Nanopore Technology (ONT) as per document no. PTC_9096_V109_REVf_06fEB2020. This included End Repair/dA tailing, Native Barcode Ligation, and Adapter Ligation of the PCR amplicons. One hundred nanograms of the pooled and purified library was sequenced using ONT's MinION Mk1B platform.

#### Phylogenetic and Comparative genomic analysis

Samples were base called and demultiplexed using Guppy basecaller (https://community.nanoporetech.com). Reads having phead quality score <7 were discarded to filter the low-quality reads. The resulting fastq files were normalized by read length (300–500) and reads were aligned using Minimap2 (v2.17) (7) to the reference (MN908947.3). Variants were called using Nanopolish (8) from the aligned reads and further creating consensus fasta using bcftools (v1.8) (9). Assembled fasta files from the SARS CoV-2 were aligned using CLC workbench and a UPGMA tree was constructed using default parameters. A secondary tree was generated after downloading whole genome sequences from VIPR (10) database from India submitted during the period from March 2020 to June 2020. Phylogenetic Analysis was done on all the compiled datasets using Vipr.

#### Lineage Analysis

Further, the assembled SARS-CoV-2 genomes were assigned lineages using the package Phylogenetic Assignment of Named Global Outbreak LINeages (PANGOLIN) (11).

#### Protein-Based Annotation

In order to categorize the specific amino acid variants present, the genomes were annotated by SnpEff version 4.5 (12). NC_045512 was taken as the reference genome of SARS-CoV-2 (13). The synonymous variants were filtered out from the analysis. The global frequency data for these 12 unique missense variations present across the four pairs was taken from cov-GLUE database which lists amino acid changes observed in GISAID SARS-CoV-2 sequences (14, 15). Total number of GISAID sequences retrieved at the time of analysis was 82,927, out of which 75,734 passed the exclusion criteria of CoV-GLUE.

#### Role of the Funding Source

The funder of the study had no role in study design, data collection, data analysis, data interpretation, or writing of the report. The corresponding author had full access to all the data in the study and had final responsibility for the decision to submit for publication.

## Results

**Figure 1:**
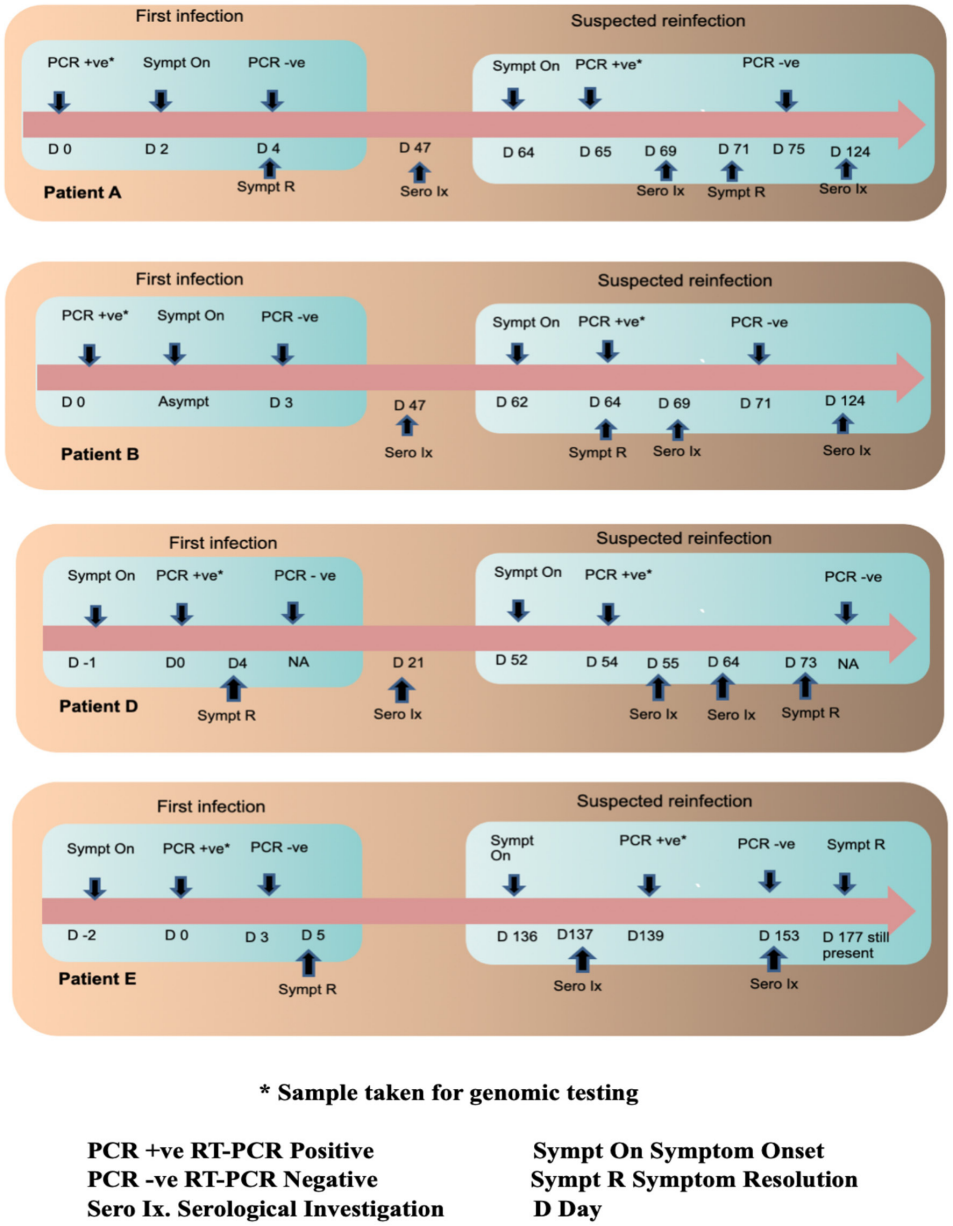
Timeline of infections in the four patients along with their serological and RT PCR investigations.

A timeline summary of the clinical presentation during the two episodes, RT-PCR testing and serology are provided in Figure 1.

### Clinical Analysis Reveals Increased Severity in the Second SARS-CoV-2 Episode

The four patients included in the study were assigned the IDs of Patient A, Patient B, Patient D and Patient E and their follow up samples were suffixed with f/u after each ID. Patient A (aged 27, male), B (aged 31, male) and D (aged 24, female) had no history of pre-existing illnesses or immunodeficiency. They were all directly involved in the clinical care of COVID-19 patients. Patient E (aged 51, female) was a controlled hypertensive, had no history of other pre-existing illnesses or immunodeficiency and worked as a technician in a COVID-19 diagnostic laboratory. Patients A and B were tested as part of a contact tracing exercise. Patient A developed sore throat and rhinitis 2 days after testing positive and recovered completely in 2 days. Patient B remained asymptomatic. Counting from their first positive RT-PCR tests A and B tested RT-PCR negative 4 and 3 days later, respectively. On day 64 and 62, respectively, they both developed COVID-19 like symptoms. Patient A tested RT-PCR positive on day 65 (1 day after symptom onset) and patient B on day 64 (2 days after symptoms onset). Patient A had fever, cough, myalgia and fatigue that lasted a week while Patient B had myalgia that lasted 2 days. Patient D's first episode was symptomatic and she tested RT-PCR positive a day after symptom onset. Symptoms included sore throat, rhinitis, and myalgia and lasted 5 days. Counting from the first positive RT-PCR, Patient D developed symptoms compatible with COVID-19 on day 52 and 2 days later on Day 54 tested positive by RT-PCR. Symptoms included sore throat, rhinitis, cough, fever, myalgia, and fatigue. Most symptoms resolved in 3 weeks but fatigue persisted for over a month. Patients A, B, and D were hospitalized during both episodes for isolation and monitoring. All three had normal respiratory rates, pulse oximetry and chest X-rays during both episodes. During the first episode, Patient E developed cough, fever, myalgia, and tested RT-PCR positive 2 days after symptoms onset. Fever remitted in 5 days but fatigue persisted. Counting from the first positive test, on day 3, RT-PCR was negative. On day 136, Patient E developed symptoms compatible with COVID-19 and 3 days later on day 139, tested RT-PCR positive. Symptoms included fever, cough, breathlessness, myalgia, nausea, and abdominal pain. Fever lasted 8 days, but breathlessness on exertion and fatigue persisted for more than 6 weeks. She was hospitalized for isolation and monitoring in the first episode but was managed as an outpatient during the second episode. Her respiratory rate and pulse oximetry were normal during both episodes but a HRCT of the chest during the second episode demonstrated pneumonia and pulmonary fibrosis. In all four patients, the second episode was more symptomatic and lasted longer in duration. All four reported that their second episodes were subjectively worse.

RT-PCR samples were collected within 3 days of symptom onset for all patients during both episodes. Patient A's sample was collected 2 days before and 1 day after symptom onset in the first and second episodes, respectively. Patient B was asymptomatic during the first episode and the sample was collected 2 days after symptom onset in the second episode. Patient D's samples were collected 1 day and 2 days after symptom onset in the first and second episodes, respectively. Patient E's samples were collected 2 days and 3 days after symptom onset in the first and second episodes, respectively. Similar time points of sample collection for the first and second episodes for the patients along with harmonized RT-PCR sample collection, processing and testing methodology allowed us to compare Ct values despite the short window for RT-PCR positivity in some COVID-19 patients. Patients A, D, and E had lower Ct values in the second episode compared to the first. Patient B's Ct values were higher during the second episode. Details of Ct values are presented in Table 1.

### Seroconversion Detected After the Second Episode

Three serological tests performed, anti-N IgG, anti-S1 RBD IgG, and neutralizing antibodies by sVNT. Counting from the first positive RT-PCR test, on day 47 Patients A and B were both negative for anti-N IgG antibodies. Their plasma samples drawn on day 47 were not stored for additional tests (which became available later). On day 69 both patients had already developed symptoms for the second time and serological sampling was repeated. Patient A became symptomatic 5 days prior and RT-PCR positive 4 days prior to serological sampling. Patient A's sample was sVNT was positive but anti-N and anti-S1 RBD IgG were both negative. Patient B became symptomatic 7 days prior and RT-PCR positive 5 days prior to serological sampling. All three serological tests were negative on day 69. A third sample was drawn for both A and B on day 124. All three serological tests were positive for Patient A. Patient B was positive by sVNT but negative for anti-N and anti-S1 RBD IgG. Counting from the first positive RT-PCR, on day 21 Patient D was negative for all three antibodies. On day 55, just 3 days after symptom onset and 1 day after RT-PCR positivity in the second episode, Patient D was positive for all three serological tests. A longitudinal sample collected on day 73 was more strongly positive for all three tests. Counting from the first positive RT-PCR test, Patient E tested negative for all three antibodies on day 137 (1 day after symptom onset in the second episode). On day 153 (17 days after symptom onset in the second episode) Patient E was positive for all three antibodies.

### Genome Analysis Reveals Clade Change and/or Distinct Mutations in the Virus Populations Between Episodes

**Figure 2:**
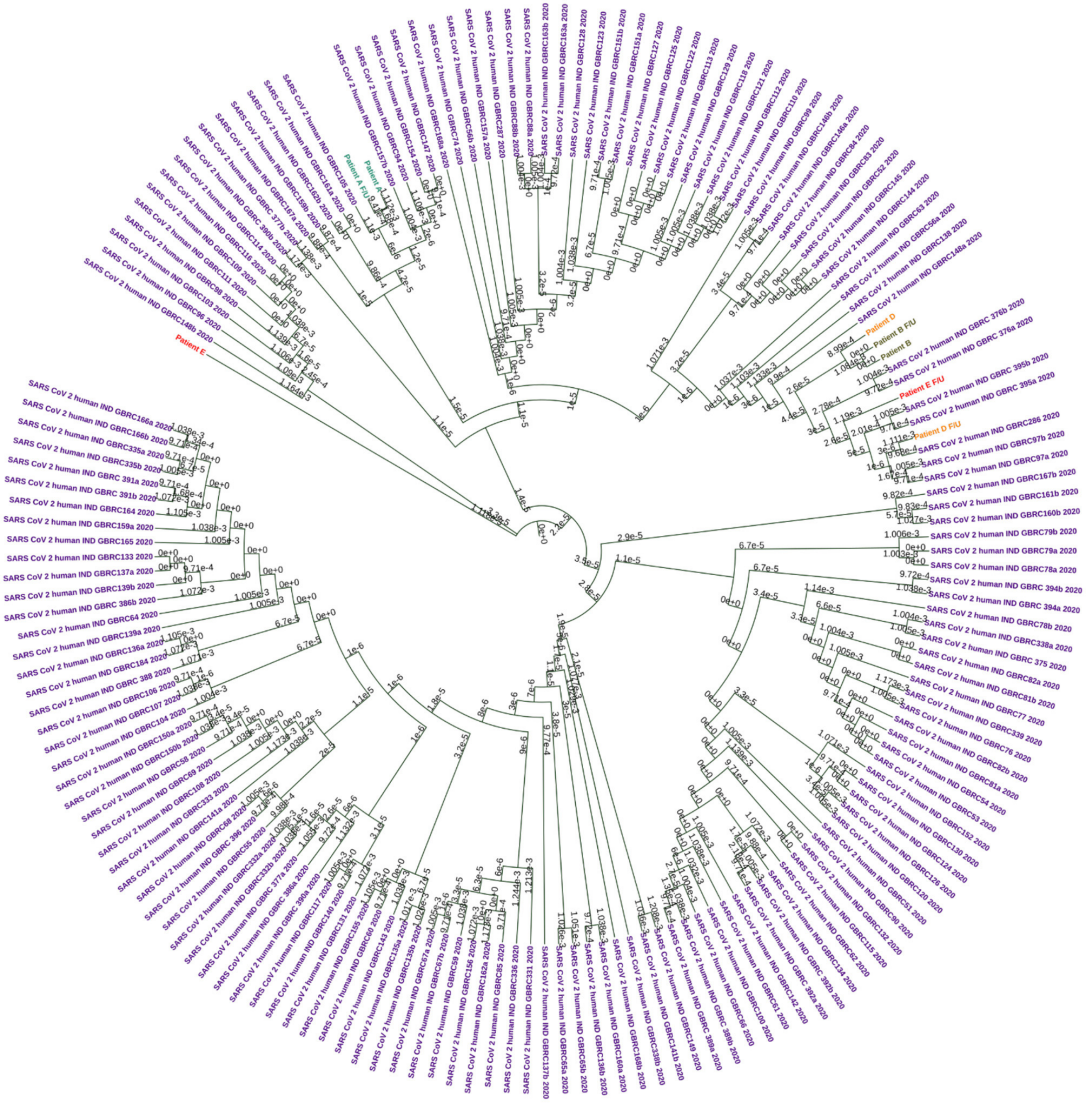
Circular Phylogram generated using UPMGA on MEGAX. A total of 160 sequences were used in the analysis. Each patient sample pairs are colored. Patient A and f/u jade green, Patient B and f/u olive green, Patient D and f/u orange, and Patient E and f/u red. Sequences downloaded from the public database are colored in purple.

Genome sequencing generated genome coverage of 80.07–99.7% (Table 2).The assembled genomes were curated and taken for further analysis. Phylogenetic tree analysis of the eight sequences, along with 160 complete viral genome sequences submitted from India in GISAID between the months of May to September 2020 because both phases of samples used for the study has been collected in this duration, revealed two samples (Patients A and B) sub-clustered together with their f/u samples respectively while samples Patient D and E and their f/u sequences clustered in different clades (Figure 2).

**Table 2:** Clade, lineage of patients with reinfections (*n* = 4).

**Sequence name**	**GISAID ID**	**Genome coverage**	**Sequencing depth**	**Lineage (PANGOLIN)**	**Most common countries (PANGOLIN)**	**Nextclade**	**GISAID**
Patient_A	EPI_ISL_528419	80.37	220	B	UK, China, USA	20A	Other
Patient_A_f/u	EPI_ISL_528420	83.01	270	B.1.80	India, Australia, Luxembourg	19A	Other
Patient_B	EPI_ISL_528421	97.78	1345	B.1	UK, USA, Australia	20B	G
Patient_B_f/u	EPI_ISL_528422	90.87	351	B.1	UK, USA, Australia	20B	Other
Patient_D	EPI_ISL_528425	85.22	311	B.1	UK, USA, Australia	19A	Other
Patient_D_f/u	EPI_ISL_528426	98.26	2299	B.1.1.32	India, UK, Spain	20B	GR
Patient_E	EPI_ISL_801538	83.99	376	B.1.5	UK, USA, Australia	19A	Other
Patient_E_f/u	EPI_ISL_676509	90.16	1233	B.1	UK, Brazil, Finland	20A	G

Clade based analysis revealed that two of eight sequences belonged to the G clade while one sequence belonged to clade GR while the remaining five sequences categorized under “Other” category. Further, analysis of lineage by PANGOLIN revealed distribution of the eight with variations of B lineages including B, B.1, B.1.80, and B.1.1.32 (Table 2).

The samples from the first and second episode of infection of the four patients are predominantly from the SARS-CoV-2 clade 19A and 20A. The clades from the first and second episode, respectively, were 20A and 19A in Patient A, 20B and 20B in patient B, 19A and 20B in Patient D, and 19A and 20B in Patient D.

**Figure 3:**
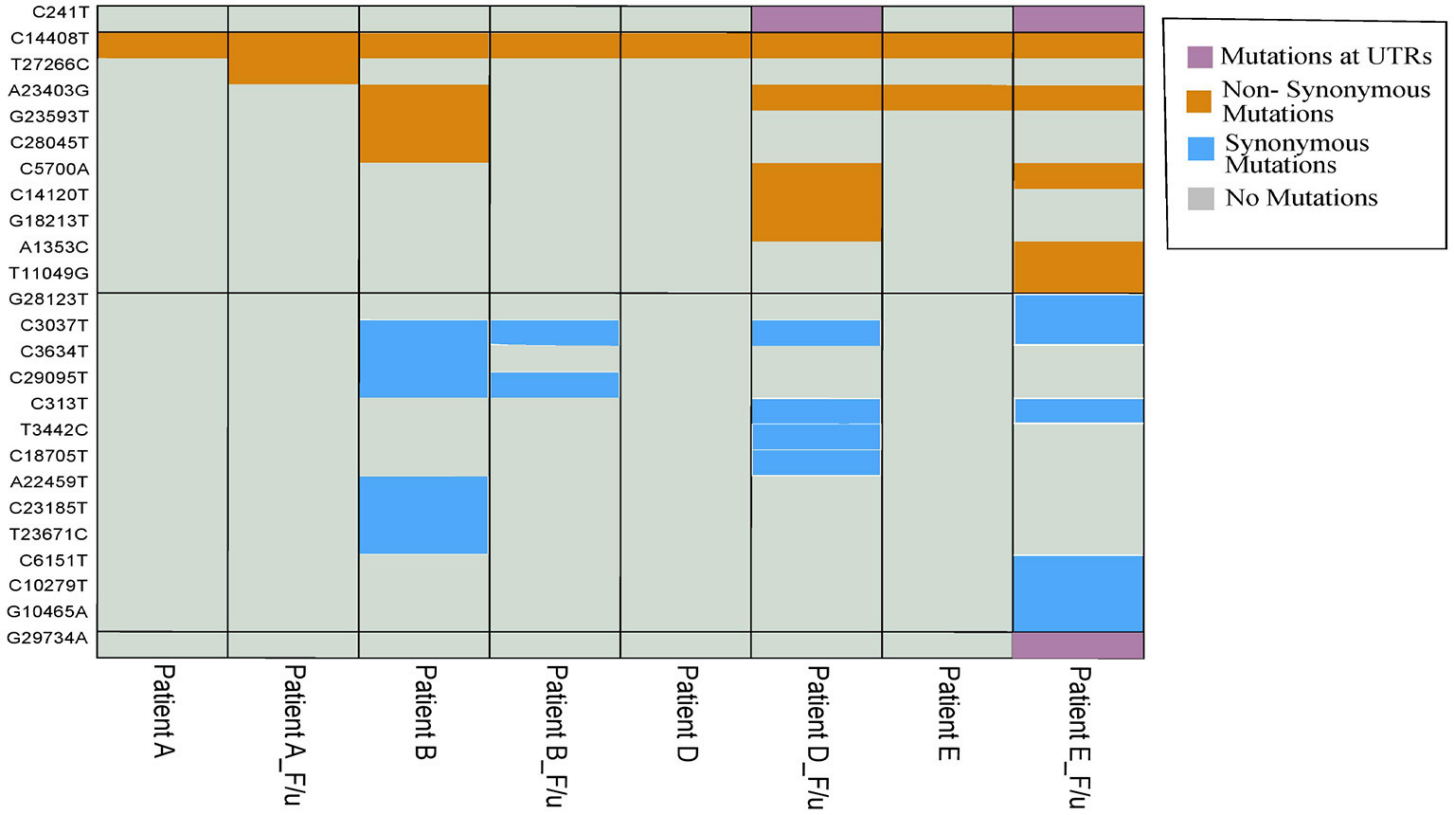
Heatmap with 42 overall mutations (unique set of 25 mutations). Purple, mustard yellow, and sky blue colors show the presence of mutation in samples while gray color shows the absence of mutation in samples.

Mutation analysis of the samples revealed distinct mutations in all the samples (Table 2). Interestingly, we observed a higher number of mutations in the follow-up samples except Pair-B, which had 10 mutations in first infection compared to three in the follow-up. Pair-E had the highest number of 13 mutations in the follow-up sample compared to two in the first sample, followed by Pair-D with 10 mutations in follow-up and one in the first sample and lastly by Pair-A with two in follow-up and one in the first sample. A total of 42 (Figure 3) mutations were observed in our sample set of four patients. Twenty-two non-synonymous, 17 synonymous, and 2 upstream UTR and 1 downstream UTR mutation is observed. Interestingly the non-synonymous mutation P323L in the nsp 12 RNA-dependent RNA polymerase gene has been reported to be concurrently present with D614G mutation in the spike protein, is observed in all patient samples, whereas D614G mutation was observed only in four samples (16, 17). In the nsp3 region, part of the replicase complex, two synonymous mutations F924F, N1123N, and one non-synonymous mutation A1812D observed in mild cases of COVID-19 (18) were observed in Patient E, Patient B, and Patient B f/u samples, respectively.

**Figure 4:**
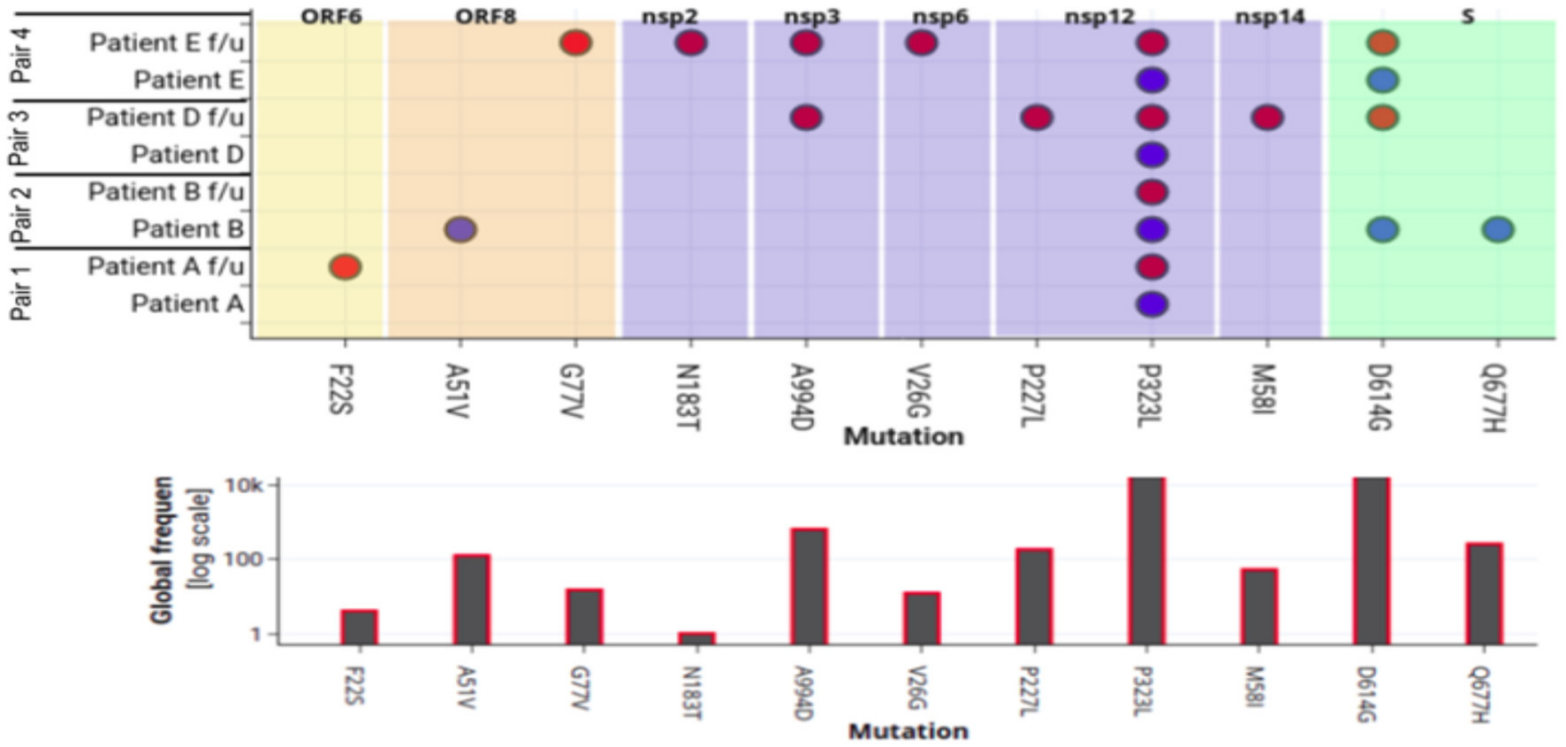
Mapping of amino-acid substitutions within SARS-CoV-2 genome of four pairs of samples. The upper plot demonstrates the seven proteins in different colors that harbor 12 non-synonymous mutations shown in dots. The Y-axis shows the four pair of patient samples. The blue and red dot indicates the presence of the mutation in the first and second episode of infections respectively. The lower plot shows the frequency of that particular mutation in 82,927 genomes deposited in GISAID.

To evaluate amino-acid alterations, we performed protein-based annotation of the 22 non-synonymous mutations found from our genome analysis of the four pair of samples (Figure 4). It was observed that Pair 1, i.e., Patient A shows minor variations, with common ones occurring within Nsp12. With respect to the other patients, interestingly, we found heterogeneity within mutations in both episodes. For instance, in Patient B, the mutations within Spike protein (D614G, Q677H) in the first episode were missing in the followup sample. Similarly, in Patients D and E, we found presence of additional mutations in samples of followup. Interestingly, in re-infection cases, a higher number of mutations were found in non-structural proteins, including nsp1, nsp2, nsp3, nsp5, nsp6, and nsp12, and nsp 14. Further, we also performed correlations of these mutations with viral genomes from world-wide populations (~1,44,426) to understand their relative frequency (Figure 2). While P323L mutation within nsp12 was found in all samples without exception, other frequent mutations showed abrupt patterns. In particular, D614G mutation within the Spike protein was consistently present in both infections in Patient E but was present only in one of the episodes in Patients B and D.

### Sequence Submission

All SARS-CoV-2 sequences from eight patients were submitted to GISAID under the accession number EPI_ISL_528419 and EPI_ISL_528420 for patient A, A_f/u, EPI_ISL_528421, and EPI_ISL_528422 for patient B, B_f/u, EPI_ISL_528425, and EPI_ISL_528426 for patient D, D_f/u, EPI_ISL_801538, and EPI_ISL_676509 for patient E, E_f/u.

## Discussion

Clinically SARS-CoV-2 infection can present with or without symptoms and severity has been categorized into four types ranging from asymptomatic to critical illness based on symptoms, clinical findings, chest imaging and blood gases as presented in Supplementary Figure 1 (19). New immunological evidence is enriching our knowledge of the immune response to infection (20) and duration of immunity following infection (21). Emerging evidence suggests Ct values and viral loads at the time of diagnosis maybe implicated in pathogenesis and disease severity (22). A handful of confirmed SARS-CoV-2 reinfection have been published on the basis of genome variation observed in the viruses between the two episodes with varying clinical manifestations between the episodes (2, 3, 23, 24). The European Center for Disease Control and Prevention (ECDC) (25) and United States Center for Disease Control and Prevention (US CDC) (26) have considered multiple criteria to investigate a case of suspected reinfection.

On the basis of these criteria, we discuss our patients and confirm or reject a case as SARS-CoV-2 reinfection. As per the US CDC, SARS-CoV-2 reinfection should be considered in individuals with COVID-19 like symptoms and a positive RT-PCR for SARS-CoV-2 with a Ct value <33 at least 45 days after the first positive RT-PCR. There should not be an obvious alternative etiology for the symptomatic second episode. Paired samples from the two episodes should undergo genomic testing that includes evaluation of single nucleotide variations (SNV) and clades to distinguish between viral persistence within host evolution vs. reinfections. In patients meeting the above criteria, genomic testing revealing differing clades as defined in Nextstrain (27) and GISAID of SARS-CoV-2 between the first and second infection is considered the best evidence of SARS-CoV-2 reinfection. More than two nucleotide differences per month in consensus between sequences that meet quality metrics is considered moderate evidence. The US CDC also recommends serial serological testing.

Accordingly our present study evaluates clinical, RT-PCR, genomic and serological information to evaluate reinfections in four patients who presented with repeat episodes of SARS-CoV-2 infections. Of the four patients in the study, Patients A, D, and E had COVID-19 like symptoms during both first episodes and second episode and did not have an obvious alternate etiology for their COVID-19 like symptoms. Their symptoms were also accompanied by a positive RT-PCR for COVID-19 over 45 days from the first positive RT-PCR. Interestingly, Patients A, D, and E had increased clinical severity and lower Ct values in the second episode. All three had Ct values not exceeding 23. Such Ct values correlate with active viral replication and positively correlate with virus culture positivity (28). Analysis of whole genome sequence data generated from the samples of both episodes of Patients A, D and E revealed that the two paired samples clustered in different clades and belonged to different lineages.

Patient A's paired samples contained viruses from different clades but were separated by a single mutation. Moreover, the sample from the second episode had low Ct values (23 in confirmatory gene) and the clinical picture strongly suggested active SARS-CoV-2 infection. Crucially, Patient A was positive for neutralizing antibodies just 5 and 4 days after symptom onset and RT-PCR positivity during the second episode. While WGS showed a single distinct mutation in consensus sequences, the clinical picture, low Ct values, difference in clade and presence of neutralizing antibodies within 5 days of symptom onset supports reinfection. It should be noted that Patient A's first sample genome coverage was 80.37 and in the second episode was 83.01. This could have resulted in detection of fewer mutations. Despite the clade change, clinical picture, lower Ct values, and nAb positivity, with the caveat of genomic coverage and based on the CDC criteria for defining reinfection, we determined the evidence as weak evidence for assigning the second episode of Patient A as a reinfection.

Patient B was asymptomatic in the first episode and but had a symptomatic second episode about 2 months later with myalgia and malaise. The Ct value from samples for RT-PCR was 33 in the first episode but 36 in the second episode. The genome analysis of the paired samples of this patient further showed no clade or lineage difference. However, mutation analysis revealed difference in mutations observed including the presence of the D614G mutation only in the sample from the first episode. There were addition/deletion of both synonymous and non-synonymous mutations between the samples of the two episodes as was observed in the functional protein annotation analysis. Most of the mutations were found in the spike protein, the region most likely to undergo mutations to escape immune pressure during prolonged infections. Three synonymous and two non-synonymous mutations occurred in the spike region. Additionally, in the second episode, 7 and 5 days after symptom onset and RT-PCR positivity all three antibody tests (anti-N, anti-S1 RBD, and sVNT) were negative. All these analyses put together make it difficult to differentiate between a prolonged infection and a reinfection in Patient B.

Both patient D and E had symptoms compatible with COVID-19 during both episodes and the clinical picture was strongly suggestive of COVID-19. Both had lower Ct values in the second episode suggestive of active viral replication. Additionally, during the second episode Patient E had radiological evidence of acute pulmonary infection (pneumonitis) superimposed on COVID-19 pulmonary sequelae (pulmonary fibrosis). Paired samples from both Patient D and E contained viruses from different clades and had distinct mutations exceeding the cut off requiring >2 distinct mutations per month between consensus sequences clearly confirming SARS-CoV-2 reinfection.

In the present study, we found priming of immunity in the first episode leading to a boosting effect following the second episode by production of neutralizing antibodies early in the second episode. Analysis of the serological profiles of all the patients failed to reveal seroconversion after the first episode but during the second episode, neutralizing antibodies were detected 5 and 3 days after symptom onset as seen in Patients A and D, respectively. Further, longitudinal samples of these patients revealed increasing titers of neutralizing antibodies. In the case of Patient E, seroconversion was not detected early in the second episode but was observed two and a half weeks after symptoms onset. While most individuals do seroconvert following SARS-CoV-2 infection, some individuals do not seroconvert (20). It is possible that the patient sin our study had failure of humoral immunity which may explain the absence of detectable antibodies. It is possible that the absence of seroconversion predisposed them to reinfection.

While our study found that the second episode was more symptomatic with a longer duration of illness, our study was not designed to identify reasons for increased severity in the second episode. Nevertheless, we hypothesize a few possible reasons for the observed increased severity in the second episode.

Some evidence from animal studies suggests that increased inoculum size or a higher infecting dose may result in increased clinical severity (29). Owing to their status as health care workers caring for COVID-19 patients or handling their samples all four patients had an occupational risk of exposure. It is possible that the participants in our study were exposed to a larger infecting dose in the second episode as compared the primary infection. Another aspect to consider is the impact of mutations in the viral genome. Recent detection of SARS-CoV-2 variants has raised important questions about the impact of S gene mutations and deletions on increased transmissibility, ACE-2 receptor affinity, viral loads, immune escape, and severity. S variants of SARS-CoV-2 have been associated with significantly lower median Ct values suggesting that changes in the S protein RBD may result in increased viral loads (30). While our sample size and absence of viral culture studies does not allow us to make determinations about the impact of S gene mutations and deletions on clinical severity and viral load, it is possible that mutations at the Spike gene may explain lower Ct values and increased severity in the second episodes.

Some experimental *in vitro* studies suggest the possibility of antibody dependent enhancement of SARS-CoV-2 (31, 32) which has also been observed in other coronaviruses. It is possible immune enhancement may have increased the severity of the second episode.

Taken altogether, our present study provides a level of evidence classified by US CDC as best evidence of reinfection in two patients (Patients D and E), weak evidence with possible reinfection in one patient (Patient A), and we were unable to differentiate between prolonged infection and reinfection in the case of Patient B. Our study adds to the growing body of evidence of SARS-CoV-2 reinfections and demonstrates the value of serial serological data in supporting reinfection claims. Our study highlights that SARS-CoV-2 reinfections do occur, and individuals who have recovered from SARS-CoV-2 infection should continue to take infection prevention precautions.

## Data Availability Statement

The datasets presented in this study can be found in online repositories. The names of the repository/repositories and accession number(s) can be found in the article/Supplementary Material.

## Ethics Statement

The studies involving human participants were reviewed and approved by Institutional Review Board of Kasturba Hospital of Infectious Diseases; IRB number 015/2020. The patients/participants provided their written informed consent to participate in this study.

## Author Contributions

JS conceptualized and designed the study. JS and LP identified the study participants. SP and SA collected and compiled data from different sources. NC and MP performed RNA extraction, aliquoting, and RT-PCR. RP, VA, JSV, AK, RM, and SF performed genome sequencing. RP, VA, JSV, AK, RM, SF, LT, SS, SC, and CS performed genomic and linage analyses. SP, SS, and JS drafted and revised the manuscript. JS, SS, and AA provided resources and participated in overall supervision. All authors contributed to data interpretation, critically reviewed the manuscript, provided contributions to tables, figures and text in the manuscript, and approved the final manuscript for submission.

## Conflict of Interest

The authors declare that the research was conducted in the absence of any commercial or financial relationships that could be construed as a potential conflict of interest.
