# Supplementary material for: Clinical, Serological, Whole Genome Sequence Analyses to Confirm SARS-CoV-2 Reinfection in Patients From Mumbai, India
Source: Front Med (Lausanne). 2021 Mar 9;8:631769. doi: 10.3389/fmed.2021.631769 (PMC7985553; doi:10.3389/fmed.2021.631769)
Supplement: Supplementary file 1 [file Data_Sheet_1.pdf]

Supplementary Material

**Patient ID**	**Sample** Nasopharyngeal (NP) Oropharyngeal (OP)	**Collection details**	**Aliquoting and storage**	**RNA extraction and RT-PCR**
**Patient A**	NP+OP	Collected in HiViral™ Transport Kit and transported in cold chain to diagnostic lab	Samples were divided into four aliquots (1-4) and RT PCR was performed on aliquot 1. Aliquots 2-4 were stored at -80C degrees for future use.	Automated RNA extraction performed using Mylab's Maverick Magnetic Bead-based Extraction kit on KingFisher Flex Extraction System followed by multiplex real-time RT-PCR using TaqPath™ COVID19 RTPCR kit for the qualitative detection of nucleic acid of SARS-CoV-2 from Applied Biosystems.
**Patient A f/u**	NP+OP			
**Patient B**	NP+OP			
**Patient B f/u**	NP+OP			
**Patient E**	NP+OP			
**Patient E f/u**	NP+OP			
**Patient D**	NP+OP		Aliquot of extracted RNA transferred to our lab was utilised for whole genome sequencing by the sequencing group	Manual extraction was done and PCR was conducted on both times using the above kit &Xpert® Xpress SARS-CoV-2
**Patient D f/u**	NP+OP			

Supplementary Table 1: Sample collection, RNA extraction, RT-qPCR, aliquots

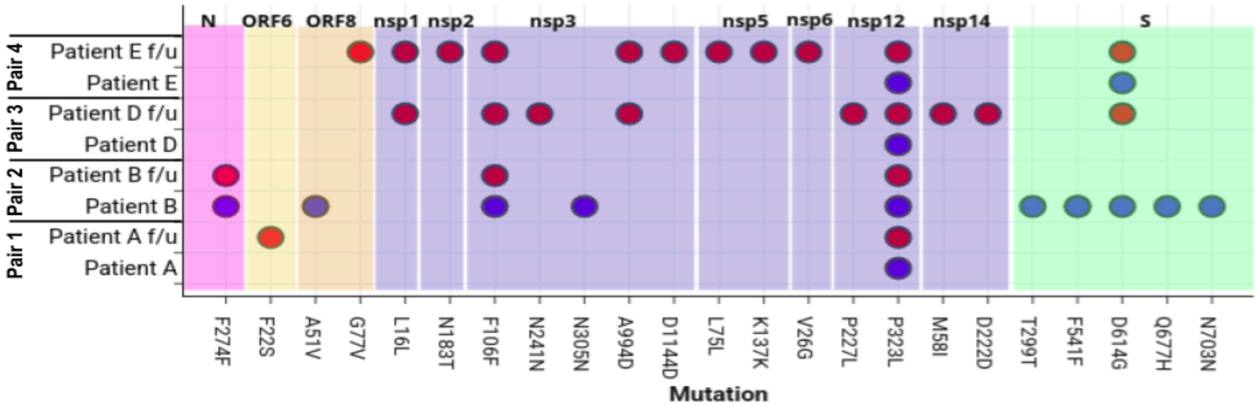

Supplementary Figure 1: Mapping of amino-acid substitutions within n-SARS-CoV-2 genome of four pairs of samples. The upper plot demonstrates the seven proteins in different colors that harbor all 39 mutations shown in dots. The Y-axis shows the four pair of patient samples. The blue and red dot indicates the presence of the mutation in the main, and re-infected cases.
